# Transfer Learning via Deep Neural Networks for Implant Fixture System Classification Using Periapical Radiographs

**DOI:** 10.3390/jcm9041117

**Published:** 2020-04-14

**Authors:** Jong-Eun Kim, Na-Eun Nam, June-Sung Shim, Yun-Hoa Jung, Bong-Hae Cho, Jae Joon Hwang

**Affiliations:** 1 Department of Prosthodontics, Yonsei University College of Dentistry, Yonsei-ro 50-1, Seodaemun-gu, Seoul 03722, Korea; gomyou@yuhs.ac (J.-E.K.); jennynam90@yuhs.ac (N.-E.N.); jfshim@yuhs.ac (J.-S.S.); 2 Department of Oral and Maxillofacial Radiology, School of Dentistry, Pusan National University, Dental Research Institute, Yangsan 50610, Korea; yhjung@pusan.ac.kr (Y.-H.J.); bhjo@pusan.ac.kr (B.-H.C.)

**Keywords:** implant fixture classification, artificial intelligence, deep learning, convolutional neural networks, periapical radiographs

## Abstract

In the absence of accurate medical records, it is critical to correctly classify implant fixture systems using periapical radiographs to provide accurate diagnoses and treatments to patients or to respond to complications. The purpose of this study was to evaluate whether deep neural networks can identify four different types of implants on intraoral radiographs. In this study, images of 801 patients who underwent periapical radiographs between 2005 and 2019 at Yonsei University Dental Hospital were used. Images containing the following four types of implants were selected: Brånemark Mk TiUnite, Dentium Implantium, Straumann Bone Level, and Straumann Tissue Level. SqueezeNet, GoogLeNet, ResNet-18, MobileNet-v2, and ResNet-50 were tested to determine the optimal pre-trained network architecture. The accuracy, precision, recall, and F1 score were calculated for each network using a confusion matrix. All five models showed a test accuracy exceeding 90%. SqueezeNet and MobileNet-v2, which are small networks with less than four million parameters, showed an accuracy of approximately 96% and 97%, respectively. The results of this study confirmed that convolutional neural networks can classify the four implant fixtures with high accuracy even with a relatively small network and a small number of images. This may solve the inconveniences associated with unnecessary treatments and medical expenses caused by lack of knowledge about the exact type of implant.

## 1. Introduction

Since Professor Brånemark’s introduction of the concept of osseointegration in the 1960s through preclinical and clinical studies, implant dentistry has developed rapidly, becoming a common treatment for tooth loss [1,2,3]. Starting from basic machined surface implants, various surface treatment methods, such as resorbable blasting and sandblasted large-grit acid-etching, have been developed, and the threads and platform shapes of implants have continued to evolve with slight improvements [4,5,6]. At present, the survival and success rates of these improved implants are very high in a wide variety of clinical situations, including systemic diseases and cases posing limitations in bone quality and volume at the implantation site [7,8,9,10]. Thus, dental implants show much better long-term stability compared to conventional fixed partial dentures or removable dental prostheses, with many studies reporting survival rates of more than 95% for dental implants [11,12].

Continued developments in this area have led to the availability of a variety of implant systems in the market in recent years [13,14,15]. Implant systems are selected and placed according to the preferences and familiarity of clinicians, as well as the masticatory force, bone quality, bone volume, and restoration space available in the patient’s tooth loss area [13,16,17,18]. With time, some of the older implant varieties have been discontinued and their production ceased, while many new types of implants, which are considerably different from the existing implant fixtures, have been introduced by the same company. Moreover, clinicians’ preferences for implant systems change over time. Jokstad et al. [15] reported the existence of approximately 220 implant brands from 80 companies worldwide. Even so, the number of implant brands in the market has increased since the publication of this study.

These developments are important because as the types of implants being used have changed over time, knowledge about these implant systems and their inter-compatibilities need to be updated for the current generation of working clinicians [19,20]. The younger generation of clinicians may lack experience with implant systems used 20 to 30 years ago, and it may be difficult for certain dentists to identify new implant systems simply by viewing the images of the fixtures in radiographs. For this reason, it can be difficult to find the most suitable replacement for a screw even when common complications occur with the implants, such as screw loosening and screw fractures. This could cause many difficulties in clinical situations, requiring new prosthetics to be manufactured. Then, it is possible that implants may no longer be maintained as required because new prostheses may not be available or other complications may arise, although no issues exist with regard to the osseointegration of the implant fixtures and the surrounding alveolar bone. In the absence of other medical records, knowledge about the type of implant would be revealed only by relying on radiographs because most parts of implant fixtures are buried in the alveolar bone, which cannot be observed in oral examination. Thus, radiographic identification of implants is especially important to provide appropriate diagnoses and treatments to patients.

Research has also been conducted to develop and evaluate implant recognition software (IRS) via creation of a database and classification of the features of implant systems fulfilling the same functions [14]. However, the database lists the characteristics of the implants based on the information provided by the implant manufacturer in the brochure. Therefore, to identify the desired implant, the details in each of the nine drop-down menus, including implant type, thread feature, surface, and collar details, must be entered manually. Moreover, the software cannot directly analyze images.

Artificial intelligence (AI) has come to play a crucial role in healthcare in recent times. In particular, convolutional neural networks (CNNs) are excellent for the detection of breast cancer, skin diseases, and diabetic retinopathy through the study of medical images [21,22,23]. CNN is the most essential algorithm for current deep learning, which is driving AI development in recent years. CNN is particularly useful for finding patterns to recognize objects and scenes in an image. CNN learns directly from the data, using patterns to classify images without the need to manually extract features. [24,25] In the dental field, AI is widely used for the detection of dental caries, measurement of alveolar bone loss due to periodontitis, numbering of teeth through tooth shape recognition, and detection of the inferior alveolar nerve [24,25,26,27,28]. 

Transfer learning with pre-trained networks has been used for high accuracy and generalization. Transfer learning is also effective for applying learned features from large datasets to small datasets to raise their accuracy and performance. In this study, five popular pre-trained networks in the Pareto frontier were applied for implant type classification while considering the accuracy and computational burden [29,30]. The Pareto frontier comprises all networks that outperform the other networks on both metrics considered for comparison (in this case, accuracy and prediction time). Deeper networks can generally achieve higher accuracy by learning richer feature representations. However, deep networks such as Xception and DenseNet require larger amounts of computing power and are characterized by longer prediction times when using graphic processing units (GPUs), but this aspect is difficult to comply with in average research and clinical environments. 

Therefore, in this research, we find the optimal pre-trained network architecture that satisfies both the accuracy and the computing power requirements for the classification of implant fixture periapical radiograph images. The tested networks were SqueezeNet, GoogLeNet, ResNet-18, MobileNet-v2, and ResNet-50.

## 2. Materials and Methods

### 2.1. Ethics Statement

This study was approved by the Institutional Review Board (IRB) of the Yonsei University Dental Hospital (Approval number: 2-2019-0068). The IRB waived the need for individual informed consent, and thus, a written/verbal informed consent was not obtained from any participant, as this study featured a non-interventional retrospective design and all the data were analyzed anonymously. 

### 2.2. Materials 

In this study, we used images of 801 patients (aged 19–84 years) undergoing intraoral x-rays (Carestream RVG 2200 intraoral x-ray system with 6100 sensor, carestream dental, Rochester, NY, USA) using paralleling technique with 60 kVp, 7 mA, and 0.08~0.1 sec between 2005 and 2019 at Yonsei University Dental Hospital. Images containing four types of implants were selected for this work: Brånemark Mk TiUnite, Dentium Implantium, Straumann Bone Level, and Straumann Tissue Level implants (Table 1). 

### 2.3. Methods 

#### 2.3.1. Preprocessing

**Figure 1:**
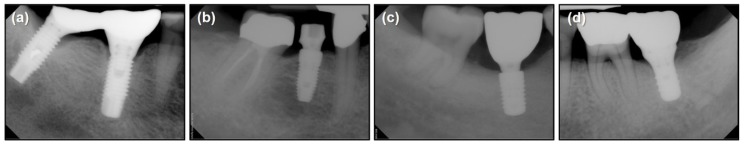
Periapical radiographs of the four types of selected implants. (**a**) Brånemark Mk TiUnite, (b) Dentium Implantium, (**c**) Straumann Bone Level, and (d) Straumann Tissue Level implants.

Images containing only one implant type were used for the network training (Figure 1). If the image presented more than one implant type, a region containing only one implant type was cropped manually. No filtering or enhancement was applied to the images, and thus, the network parameters were learned from the raw images.

#### 2.3.2. Network Pre-training 

**Figure 2:**
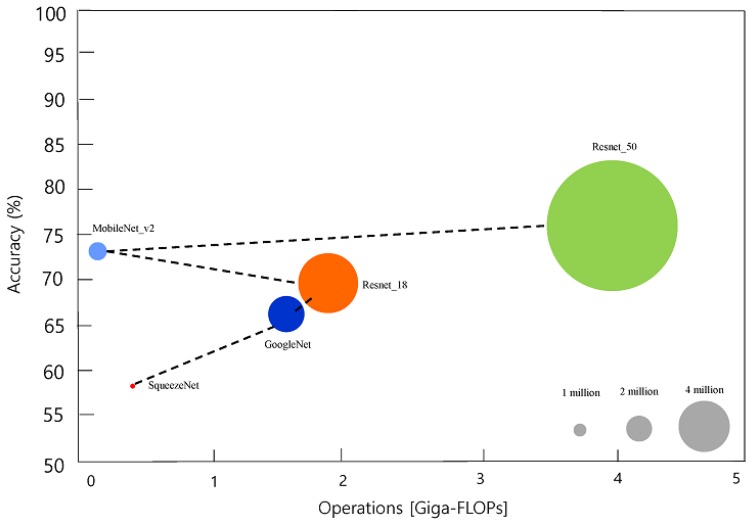
Relative speeds and accuracies of the different networks used in this study. Black dash line represents for Pareto frontier: data from Benchmark Analysis of Representative Deep Neural Network Architectures [30].

We tested from smaller to bigger network architectures in the Pareto frontier (Figure 2) [29,30]. All the pre-trained networks were trained on more than a million images from the ImageNet database [31], which can classify the images into 1000 object categories, such as keyboard, mouse, pencil, and many animals. As a result, the networks could learn rich feature representations for a wide range of images. The basic properties of the pre-trained networks are presented in Table 2.

**Figure 3:**
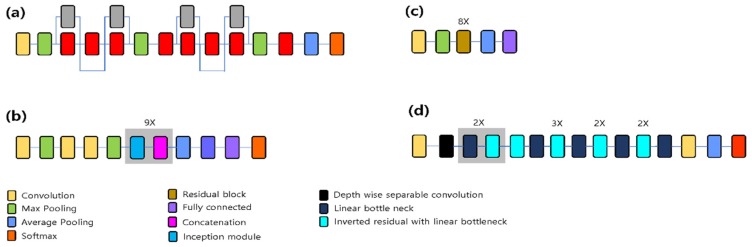
Pretrained network architectures. (**a**) SqueezeNet, (**b**) GoogLeNet, (**c**) ResNet-18, and (**d**) MobileNet-v2. In ResNet-50, each 2-layer residual block of ResNet-18 is replaced in the 34-layer net with the 3-layer bottleneck block.

SqueezeNet: The SqueezeNet architecture is introduced to show the AlexNet-level accuracy with 50× fewer parameters and model compression techniques. The network is an 18-layer-deep DAGNetwork [32]. SqueezeNet v1.1 was used in this article. It has an accuracy similar to that of SqueezeNet v1.0, but requires fewer floating point operations per prediction (Figure 3a) [33]. 

GoogLeNet: GoogLeNet introduced the inception module to extract features more effectively using various filter sizes (1 × 1, 3 × 3, and 5 × 5). This network also uses 1 × 1 convolution to reduce the network dimension and computation burden (Figure 3b) [34].

ResNet: ResNet was introduced to solve the unexpected low performance of deeper network architectures. The network uses skip connection to convey information from the previous input layer to the output layer [35]. Several variants with different output sizes exist, including Resnet-18, ResNet-34, ResNet-50, ResNet-101, and ResNet-152. In this study, ResNet-18 (Figure 3c) and ResNet-50 were used. ResNet-18 has 8 repetition of 2-layer residual block whereas ResNet-50 has 16 repetition of 3-layer bottleneck block.

MobileNet-v2: The network is 54 layers deep and is specialized toward use in mobile devices. It introduces more computationally effective convolution layers, such as the depthwise, pointwise, expansion, and projection convolution layers (Figure 3d) [36].

#### 2.3.3. Data Augmentation

Various data augmentation techniques were used to prevent overfitting due to the small dataset size. Training images were rotated from −20° to 20°, randomly horizontally rotated, translated horizontally and vertically from −30 to 30 pixels, and scaled horizontally and vertically from 0.7 to 1.2.

#### 2.3.4. Training Options

Of the 801 images, the dataset was randomly divided into the training:validation:testing sets in the ratio 6:2:2. NVIDIA Titan RTX GPU was used for the network along with MATLAB 2019b (MathWorks, Natick, MA, USA). The models were trained for a maximum of 500 epochs with the sgdm optimizer [37]. The minibatch size was 10, and the initial learning rate was 3 × e^−4^. The training process was stopped early when the validation patience values of 20, 40, 60, 80, 100, 120, and 140 (Table S1). Multiple validation patience values were used to prevent overfitting for each pre-trained network in different epochs. The model with the best performance for each pre-trained network on a validation dataset was selected. If the model showed same performance for multiple validation patience, the model of the smallest validation patience among them was selected.

#### 2.3.5. Metrics for Accuracy Comparison

The accuracy, precision, recall, and F1 score were calculated as follows with the test dataset using the confusion matrix.

(1) $$\mathrm{Accuracy}=\frac{\mathrm{TP}\;+\;\mathrm{TN}}{\mathrm{TP}\;+\;\mathrm{FP}\;+\;\mathrm{FN}\;+\;\mathrm{TN}},$$

(2) $$\mathrm{Precision}=\frac{\mathrm{TP}}{\mathrm{TP}\;+\;\mathrm{FP}}$$

(3) $$\mathrm{Precision}=\frac{\mathrm{TP}}{\mathrm{TP}\;+\;\mathrm{FN}}\;\mathrm{and}$$

(4) $$F1\;\mathrm{Score}=\frac{2\times \left(\mathrm{Recall}\;\times \;\mathrm{Precision}\right)\;}{\mathrm{Recall}\;+\;\mathrm{Precision}}$$

TP: true positive, FP: false positive, FN: false negative, TN: true negative

## 3. Results

### 3.1. Classification Performance

**Figure 4:**
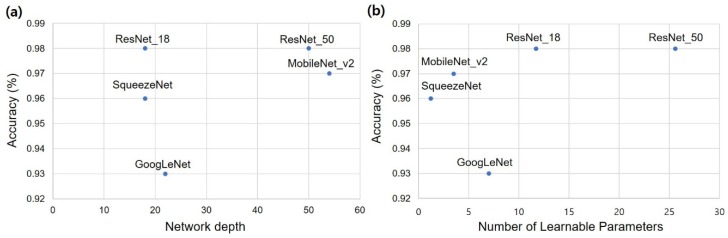
Test accuracy with respect to network depth and number of parameters. (**a**) Network depth, (**b**) Number of parameters.

The accuracy of the implant fixture system classification is described in Table 3. All five models showed a test accuracy of more than 90%. Network depth and test accuracy showed no significant trends (Figure 4a). The number of learnable parameters and test accuracy were roughly proportional, but the graph for the ResNet-18 model was already saturated (Figure 4b). SqueezeNet and MobileNet-v2, which are relatively small networks with less than four million parameters, showed an accuracy of approximately 96% and 97%, respectively.

### 3.2. Training Progress

**Figure 5:**
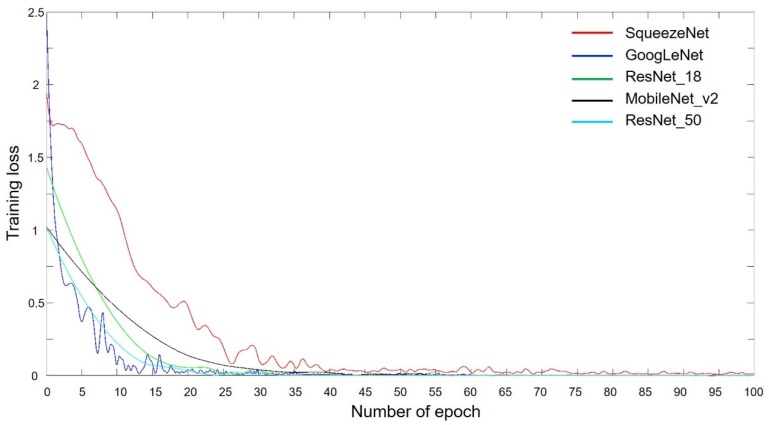
Training progress of the five pretrained networks with respect to the number of epochs.

GoogLeNet and ResNet showed a sharp decrease in training loss, while SqueezeNet exhibited a relatively slower decrease than both these networks in this regard. However, MobileNet-v2 showed the slowest training loss reduction. All the models converged within 100 epochs (Figure 5).

### 3.3. Visualization of Model Classification

**Figure 6:**
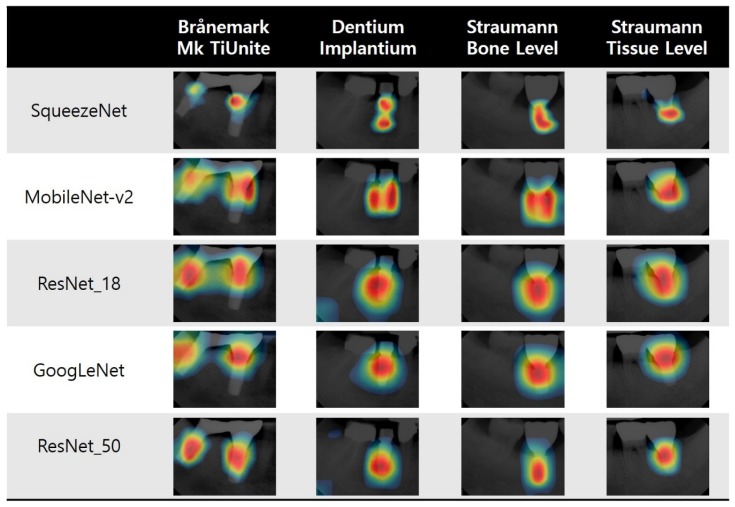
Example of the class activation maps of the five pretrained networks for the four selected implant fixture types.

A class activation map (CAM) indicates the discriminative region used by CNN to identify an image as the category [38]. In CAMs, every network searches for the implant fixture. Thus, we can visually confirm the reason for the high accuracy of all five networks. However, the parts and ranges of the fixtures that are focused on differ from one model to another (Figure 6).

## 4. Discussion

Categorizing the implant fixture type in periapical radiographs with high accuracy is important for maintenance purposes, especially in the absence of accurate medical records. However, this exercise has not been attempted owing to difficulties in image processing and feature extraction. This study shows that a deep learning algorithm can solve this problem by learning features in an end-to-end style using training image data and without the need for complicated image preprocessing. Furthermore, the visual interpretations by the trained networks provide a reasonable explanation of the high accuracy achieved by the deep learning models.

The intraoral x-ray was taken with paralleling technique for standardization. However, absolute parallel angle with implant fixture and the sensor is not possible. This minor arbitrary angle between implant fixture and the sensor contributed as data augmentation to achieve high accuracy of our models.

It is generally known that accuracy tends to increase with deep learning, because the deeper the network, the deeper the learning. This trend was not evident in the results of this experiment, and the increase in the number of network layers was not necessarily proportional to the increase in accuracy. This is because ResNet-18 showed little loss of information between layers due to features such as skip connection. Thus, it can be considered that sufficient learning was possible despite the fewer number of layers.

As the number of parameters that can be learned increased, the graph of the ResNet-18 network became saturated once the accuracy increased. SqueezeNet and MobileNet-v2, which are small networks with less than four million parameters, showed accuracies exceeding 96%. This is due to the fact that in periapical x-rays, the number of features required to classify the four implant fixtures may be small, thus, sufficient learning is possible with a relatively small number of parameters. In the case of ResNet-18, the network already showed a test accuracy close to 1, similar to the case of ResNet-50. Therefore, it appears that the current network structure requires approximately up to 10 million features to distinguish the four implant fixtures in periapical x-rays. In the future, this number can be reduced as the network structure evolves.

This study confirmed that a CNN can analyze implant images and automatically classify the four selected implant fixture types with high accuracy even with a relatively small network size and a small number of images. This means that implant classification networks can be easily learned with relatively low computing power and be applied in mobile environments, making them useful and convenient in clinical situations. Moreover, even a small number of radiographs of older implant systems can be used to create a network to distinguish the different types of implants. This may do away with unnecessary treatments and medical expenses caused by not knowing the exact type of implant. The results of this study may also help in the development of decision assistance software using medical images. This study also showed that trained models search for a distinct part of each implant precisely. As per the CAMs of ResNet-50 and SqueezeNet, which showed the best localization, the networks searched for the connection between the fixture and the abutment for the Brånemark implants, for the overall fixation area in the Dentium and Straumann (BL) implants, and for the transgingival portion and fixture connection in the Straumann tissue level implants. All these parts are distinctive of each implant type. These findings prove that the deep learning model can identify the discriminative features of each implant type well. The CAM of each model revealed a slightly different focus, which will likely improve if the number of datasets increases and the accuracy of the model improves. 

This study also presents some limitations. First, only four types of implant systems were selected. Since many types of implants currently exist in the market and some have been in use for a long time, clinicians encounter many more types of implants in practice [15,20]. To expand the results of this study, it is necessary to build a database by collecting a wide variety of implant fixture images, including those of implant types that are rarely seen. In this study, we implemented a network that can detect and classify implants with high accuracy even with a small number (about 200) of radiographic images by using exhaustive image augmentation. Therefore, it may be not difficult to acquire adequate numbers of radiographs and create a classification network that includes the various implant systems not included in this study. 

This study employed images containing only one implant to determine the possibility of distinguishing implant fixtures. However, further research is needed to create a network that can detect implant fixtures using uncropped images, or to apply a technique to detect multiple implants simultaneously using networks such as You-Only-Look-Once (YOLO) and Single Shot multibox Detector (SSD).

In addition, we trained the network using only periapical radiographic images. As clinicians also use panorama radiographs for treatment purposes, it is necessary to construct a network that is able to learn and identify implant types using panorama radiographs.

In the future, the network should be able to not only classify implant types, but also their diameters and lengths. If the length of the implant can be detected automatically, the degree of marginal bone loss around the implant can be easily checked, which in turn can lead to the development of an algorithm to estimate the health and prognosis of the implant as well as diagnose peri-implantitis [39,40]. This could lead to the development of a clinically useful diagnostic software for implant-related complications. It is also very important to accurately identify the diameter of the implant, because this parameter is closely related to the implant connection type. Implants can have a variety of connections depending on the diameter, and because they are very different (depending on the implant system), they may have narrow, regular, or wide connections, which are closely related to the component compatibility of the implant prosthesis system [41,42]. By developing a network that accurately classifies the diameter of the implant, the implant system can be automatically identified, and it would be possible to know what components should be prepared for repair and maintenance when mechanical complications occur. Clinicians will also be able to obtain information about other implant systems that are compatible with the detected system. Even if it is difficult to obtain information about an implant system currently due to the discontinuation of its production and sales, a system can be established to help clinicians easily procure and respond to the provided information.

## 5. Conclusions

This study confirmed that the investigated CNNs could classify four implant fixtures with high accuracy despite the relatively small network and the small number of images. The CAM of each network was shown to distinguish the characteristic features of each implant fixture system. The results of this study may help clinicians and patients avoid unnecessary treatment and medical expenses resulting from not knowing the exact type of implant. To expand the results of this study, it is necessary to build a database comprising a wide variety implant fixture systems, including implant types that are rarely seen.

## Figures and Tables

**Table 1:** Implant type and data number.

Implant System	Brånemark Mk TiUnite Implant	Dentium Implantium Implant	Straumann Bone Level Implant	Straumann Tissue Level Implant
Number of data	197	193	203	208

**Table 2:** Properties of pre-trained networks.

Network	Depth	Size (Megabyte)	Parameters (Millions)	Image Input Size
SqueezeNet	18	4.6	1.24	227 × 227
GoogLeNet	22	27	7	224 × 224
ResNet-18	18	44	11.7	224 × 224
MobileNet-v2	54	13	3.5	224 × 224
ResNet-50	50	96	25.6	224 × 224

**Table 3:** Accuracy of implant fixture system classification according to the pre-trained networks in this research.

Pre-Trained Network	Test Accuracy	Precision	Recall	F1 Score
SqueezeNet	0.96	0.96	0.96	0.96
GoogLeNet	0.93	0.92	0.94	0.93
ResNet-18	0.98	0.98	0.98	0.98
MobileNet-v2	0.97	0.96	0.96	0.96
ResNet-50	0.98	0.98	0.98	0.98

## Supplementary Materials

The following are available online at https://www.mdpi.com/2077-0383/9/4/1117/s1, Table S1: Test and validation accuracy according to multiple validation patience.
