# Supplementary material for: Transfer Learning via Deep Neural Networks for Implant Fixture System Classification Using Periapical Radiographs
Source: J Clin Med. 2020 Apr 14;9(4):1117. doi: 10.3390/jcm9041117 (PMC7230319; doi:10.3390/jcm9041117)
Supplement: Supplementary file 1 [file jcm-09-01117-s001.pdf]

Table S1. Test and validation accuracy according to multiple validation patience.

	Test_Accuracy	Val_Accuracy	Precision	Recall	F1_Score
20 validation patience					
squeezenet	0.9125	0.913580247	0.913095	0.916753	0.914921
googlenet	0.9375	0.950617284	0.936842	0.946842	0.941816
resnet18	0.95	0.950617284	0.949342	0.953261	0.951297
mobilenetv2	0.925	0.962962963	0.926786	0.928409	0.927597
resnet50	0.9375	0.925925926	0.9375	0.944022	0.94075
40 validation patience					
squeezenet	0.875	0.888888889	0.87619	0.896101	0.886034
googlenet	0.925	0.962962963	0.924342	0.938765	0.931498
resnet18	0.95	0.962962963	0.950595	0.95202	0.951307
mobilenetv2	0.9125	0.950617284	0.91369	0.921294	0.917477
resnet50	0.975	0.987654321	0.975595	0.97619	0.975893
60 validation patience					
squeezenet	0.9625	0.987654321	0.963095	0.963033	0.963064
googlenet	0.925	0.913580247	0.924342	0.931783	0.928047
resnet18	0.925	0.925925926	0.925595	0.936012	0.930774
mobilenetv2	0.9625	0.987654321	0.963095	0.963033	0.963064
resnet50	0.95	0.987654321	0.950595	0.956522	0.953549
80 validation patience					
squeezenet	0.9	0.950617284	0.9	0.916667	0.908257
googlenet	0.9375	0.925925926	0.9375	0.94494	0.941206
resnet18	0.95	0.987654321	0.950595	0.953409	0.952
mobilenetv2	0.9625	0.962962963	0.963095	0.963033	0.963064
resnet50	0.975	0.987654321	0.975	0.97619	0.975595
100 validation patience					
squeezenet	0.95	0.950617284	0.95	0.956522	0.95325
googlenet	0.925	0.938271605	0.925	0.934659	0.929804
resnet18	0.975	1	0.975595	0.975	0.975298
mobilenetv2	0.9625	0.962962963	0.963095	0.963033	0.963064
resnet50	0.975	0.962962963	0.975	0.97619	0.975595
120 validation patience					
squeezenet	0.925	0.925925926	0.92619	0.933794	0.929977
googlenet	0.975	0.938271605	0.975	0.97619	0.975595
resnet18	0.9625	1	0.9625	0.965909	0.964202
mobilenetv2	0.9375	0.950617284	0.93869	0.940115	0.939402
resnet50	0.975	0.962962963	0.975	0.97619	0.975595
140 validation patience					
squeezenet	0.975	0.987654321	0.97619	0.977273	0.976731
googlenet	0.9875	0.962962963	0.9875	0.9875	0.9875
resnet18	1	1	1	1	1
mobilenetv2	0.925	0.987654321	0.92619	0.929911	0.928047
resnet50	0.975	0.975308642	0.975595	0.97619	0.975893
